# Effect of Physical Activity on Self-Concept: Theoretical Model on the Mediation of Body Image and Physical Self-Concept in Adolescents

**DOI:** 10.3389/fpsyg.2019.01537

**Published:** 2019-07-10

**Authors:** Juan Gregorio Fernández-Bustos, Álvaro Infantes-Paniagua, Ricardo Cuevas, Onofre Ricardo Contreras

**Affiliations:** ^1^Department of Didactics of Musical, Plastic and Physical Education, Faculty of Education of Albacete, University of Castilla-La Mancha, Albacete, Spain; ^2^Department of Didactics of Musical, Plastic and Physical Education, Faculty of Education of Ciudad Real, University of Castilla-La Mancha, Ciudad Real, Spain

**Keywords:** physical activity, self-concept, physical self-concept, body image, structural equation model, adolescence

## Abstract

**Objective:** The aim of this research was to study the mediation of body dissatisfaction, physical self-concept, and body mass index (BMI) on the relationship between physical activity and self-concept in adolescents.

**Materials and Methods:** A sample of 652 Spanish students between 12 and 17 years participated in a cross-sectional study. Physical self-concept and general self-concept were assessed with the Physical Self-Concept Questionnaire (CAF), body dissatisfaction with the Body Shape Questionnaire (BSQ), and physical activity was estimated with the International Physical Activity Questionnaire (IPAQ-SF). BMI was utilized as a measurement of body composition. Structural equation modeling was used to assess the results.

**Results:** The resulting models showed good fit indexes. Final model for all participants explained the 17% of the variance of body dissatisfaction, 57% of physical self-concept, and 60% of general self-concept. Physical activity had a positive and indirect effect on self-concept (*β* = 0.29, *p* < 0.01) and direct effects on body dissatisfaction (*β* = −0.26, *p* < 0.01) and physical self-concept (*β* = 0.20, *p* < 0.01). BMI had a direct effect on body dissatisfaction (*β* = 0.31, *p* < 0.01) and on physical self-concept (*β* = −0.10, *p* < 0.01) and an indirect effect on general self-concept (*β* = −0.24, *p* < 0.01). However, it was only associated with physical activity in males, playing a mediating role between physical activity and body dissatisfaction.

**Conclusion:** Physical activity can help individuals to achieve a positive self-concept and promote psychological well-being in adolescents through the improvement of physical perceptions and body satisfaction. The importance of BMI, body dissatisfaction, and physical self-concept on the configuration of the self-concept is also emphasized. Educational policymakers and Physical Education teachers should implement strategies to promote physical activity in the schools and provide a Quality Physical Education programs to increase physical activity during adolescence.

## Introduction

Mental health has become a public health issue among youth all over the world, having reached a 20% prevalence rate within this population (World Health Organization, Department of Mental Health, Substance Abuse, World Psychiatric Association, International Association for Child, Adolescent Psychiatry, and Allied Professions, 2005; Belfer, 2008). In this line, physical activity (PA) is considered as one of the most important factors in the prevention and treatment of these kinds of issues (Ahn and Fedewa, 2011). Research has shown that PA can provide physiological and psychological benefits (Brown et al., 2013) related to mental health (Penedo and Dahn, 2005), including a reduction in depression and anxiety and an increase in executive function (Davis et al., 2011), psychological well-being (McMahon et al., 2017), body satisfaction (Campbell and Hausenblas, 2009), and self-concept (Marsh and Peart, 1988). Indeed, self-concept is especially relevant during the school ages, as it is considered to be a primary educational aim due to its general effects on mental status, psychological well-being, and human behavior (Rosenberg, 1985).

Many studies have associated the practice of PA with self-concept. Meta-analyses confirmed these positive relationships in PA program-based interventions aimed at improving self-concept (Liu et al., 2015), as well as in observational studies (Babic et al., 2014). However, some results seem contradictory; many studies proved that PA is positively related to self-concept (Sonstroem, 1997; Beasley and Garn, 2013; Sani et al., 2016), while others did not report that association (Annesi, 2006; Robinson et al., 2010) or made that relationship conditional on other mediating factors. For instance, some studies found that the positive relationship between PA and self-concept relied on the kind of sport practiced (Slutzky and Simpkins, 2009; Fernández-Bustos et al., 2010), only occurred among girls (Noordstar et al., 2016) or only among boys (Altıntaş et al., 2014), or was conditional based on body mass index (BMI) (Reddon et al., 2017).

Either way, according to the multidimensional and hierarchical conceptualization of self-concept (Shavelson et al., 1976), the possible effect of PA on the self-concept is mediated by one of its domains, the physical self-concept (Sonstroem, 1997; Slutzky and Simpkins, 2009). Under this theory’s umbrella, Sonstroem and Morgan (1989), and later Sonstroem et al. (1994), proposed the exercise and self-esteem model (EXSEM) to explain how PA influenced self-esteem. According to this model, participating in physical activities increases the feelings of self-efficacy, improving the perceptions of sports competence and physical acceptance; in turn, this would foster general self-esteem. This model, besides having been empirically demonstrated by its authors, has been confirmed in other studies (McGannon and Spence, 2002; Murgui et al., 2016; Noordstar et al., 2016).

Body image (BI) is one of the most influential factors impacting psychological well-being, which determines the configuration of self-concept, particularly during adolescence (Thompson and Smolak, 2001; Fenton et al., 2010). Numerous studies focused on the idea that body satisfaction positively influences self-concept, as feeling comfortable with one’s body produces overall positive feelings (Van den Berg et al., 2010; Buchanan et al., 2013; Fernández-Bustos et al., 2015). Nevertheless, body dissatisfaction levels are high during adolescence, especially among girls (Kantanista et al., 2015).

Likewise, another matter of concern for researchers has been how PA influences BI. A large amount of scientific literature has focused on proving how sports and PA are associated with an improved BI (Añez et al., 2018) or how sports and PA-based interventions can be utilized to improve BI (Martin-Ginis et al., 2014). Various meta-analyses and reviews have been carried out on this subject and, although it is accepted that the relationship between BI and PA is positive for males and females, the moderator role of sex is not clear. Even though Hausenblas and Fallon (2006) found a greater effect among women when compared with men, other reviews failed to find gender differences (Campbell and Hausenblas, 2009; Sabiston et al., 2018). Notwithstanding, McIntosh-Dalmedo et al. (2018) did not find enough evidence to suggest that sport- and exercise-based interventions could improve BI among female adolescents. Even so, the generalization of the findings has been disproportionately focused on women. Only one study precisely focused on men and boys (Bassett-Gunter et al., 2017), and its results showed that a positive BI was associated with a greater participation in PA. Although some mechanisms are likely to be shared between males and females, it is also possible that there is the existence of unique mechanisms. For example, a study conducted by Martin-Ginis et al. (2005) identified sex differences on the mechanisms underlying changes as a result of PA. Therefore, it is necessary to develop a better comprehension of the mechanisms underlying the effects of PA on BI between males and females.

Meta-analyses and reviews limit the whole understanding of the research into this subject, as these follow a unidirectional framework where PA improves BI, with a gap in the bidirectional nature of these associations. Unfortunately, there is no explicit theory or framework to guide the research on PA and BI. Consequently, some authors have tried to work upon the basis of other models. For instance, Martin-Ginis et al. (2012, 2014) tried to operationalize the EXSEM model (Sonstroem and Morgan, 1989), which was initially designed to describe the effects of PA on self-esteem in an effort to understand how PA has an impact on BI.

In spite of the major relationships that may exist between BI, self-concept, and PA, only a few studies have analyzed them concurrently. Fraguela-Vale et al. (2016) found that the practice of PA was simultaneously associated with both better perceptions of BI and a higher satisfaction with oneself. Blanco-Ornelas et al. (2017) showed that regular PA had an indirect and positive effect on personal self-concept through the subjective importance of physical fitness and appearance. However, Fernández-Bustos et al. (2016) found a relationship between PA and self-concept, but not between PA and physical appearance among female adolescents with body dissatisfaction. In addition to these studies, which are mainly correlational, stronger theories are needed to help us to understand the relationship between the practice of PA and self-concept and BI’s role in these associations.

Therefore, this study aimed to determine the importance of PA in the configuration of self-concept in adolescents and the possible mediating role of BI and physical self-concept through a theoretical model. Additionally, we also studied the possible direct and indirect relationships between the different variables included in the model. To accomplish this objective, a new predictive model was designed by considering the EXSEM model (Sonstroem and Morgan, 1989) and a modification of Martin-Ginis et al. (2012), with the inclusion of BMI as a mediating variable. Alternative additional models were tested by sex to anticipate possible differences due to the uneven PA participation and body dissatisfaction (BD) between males and females. Moreover, adolescence is an essential period for the study of these constructs, as it is a sensitive stage for the self-concept and BI configuration and a consolidating period for lifelong PA habits.

## Materials and Methods

### Participants

A total of 652 students (296 male and 356 female), aged between 12 and 17 years, participated in this study. They represented approximately 80% of the youth population of La Roda (Spain). These were all the students who were available during the day of the data collection and whose parents had previously signed an informed consent for participation. Eighteen cases were excluded for some errors in data completion or data tabulation. Therefore, the final sample included in the data analyses consisted of 634 participants (284 male and 350 female).

This is an appropriate sample size for avoiding inaccurate estimates of standard errors and fit indexes, according to the criterion proposed by Jackson (2003) for structural equation modeling (SEM). This author suggested that an ideal sample size should meet the ratio of 20 cases per each parameter to be estimated in the model or a less ideal ratio of 10 cases per parameter. Considering that our larger model contained 24 parameters to be estimated, the range of the ideal sample size would be between 240 and 480 participants.

### Measures and Materials

#### General Self-Concept and Physical General Self-Concept

Two scales from the Physical Self-Concept Questionnaire (CAF) designed by Goñi et al. (2004) were utilized to assess the general physical self-concept (GPSC) and the general self-concept (GSC). This is the only physical self-concept questionnaire elaborated in Spanish without following any back-translation process. The questionnaire is completed by answering questions on a 5-point Likert scale (1 = false, 2 = usually false, 3 = sometimes true/sometimes false, 4 = usually true, 5 = true). Regarding the GPSC, students answered six items about their positive opinions and feelings (happiness, satisfaction, pride, and confidence) in the physical domain (e.g., “Physically, I am satisfied with myself”). With regard to the GSC, participants showed their overall satisfaction with their own selves and life by answering another six items (e.g., “I feel annoyed with myself”). The total questionnaire’s reliability coefficient (Cronbach’s alpha) for this study sample was *α* = 0.95. By scale, it reached a reliability of *α* = 0.89 for GPSC scale and *α* = 0.77 for the GSC scale.

#### Body Dissatisfaction

The Body Shape Questionnaire (BSQ) by Cooper et al. (1987), adapted into the Spanish context (Raich et al., 1996), was used to assess body dissatisfaction (BD). The BSQ measures dissatisfaction of one’s body, the fear of weight gain, physical appearance self-devaluation, the desire for weight loss, and the avoidance of situations in which one’s physical appearance could get attention from others (e.g., “Have you been so worried about your shape that you have been feeling you ought to diet?”). The BSQ is a 34-item self-administered questionnaire with a 6-point Likert scale (i.e., 1 = never, 2 = rarely, 3 = sometimes, 4 = often, 5 = very often, 6 = always). Concern about body image was determined from the total score of this questionnaire (scores >81). For this study sample, the internal consistency was *α* = 0.97.

#### Body Mass Index

A calibrated digital scale with a sensibility of 0.1 kg (Tanita UM-075) and a 2-meter altimeter (Holtex) were used to measure each participant’s weight and height, respectively. These measurements were collected by two ISAK level 1 researchers to calculate the participants’ BMI. Students were measured and weighted barefooted and wearing light clothes (no long trousers or sweaters were allowed). The body weight and height of each participant were measured twice, and the average between both was taken. To standardize the BMI values, participants were classified according to the cut-offs of the International Obesity Task Force (IOFT) (Cole and Lobstein, 2012) for their corresponding age and sex.

#### Physical Activity

The short form of the International Physical Activity Questionnaire (IPAQ-SF) was utilized to estimate PA levels. This self-reported measurement has shown reliability and validity within different contexts (Craig et al., 2003), including with the Spanish adolescent population (Aibar et al., 2016). For this study, only data on frequency and duration of both, moderate PA and vigorous PA, were considered. Light PA was therefore disregarded. With these data, a score of accumulative total weekly minutes of moderate and vigorous PA was calculated.

### Procedure

The present study followed a cross-sectional design. Participants were recruited from the entire population of high school students in a Spanish city (La Roda). Prior to data collection, we informed the participants and their parents about the research aims, procedures, risks, and benefits of the study, and we obtained the required consent from the corresponding educational boards, high schools administrations, and parents of the students. Participation was voluntary with the parents’ consent, and the anonymity of the participants and data confidentiality were guaranteed during the whole process. The study conformed to the deontological guidelines defined by the Declaration of Helsinki (Hong-Kong revision, 1989) and followed the recommendations of the Good Clinical Practice in the European Community (1 July 1991, document 111/3976/88) and the Spanish legislation on human clinical research (Royal Decree 561/1993 on clinical trials). The protocol was approved by the Ethics Committee on Human Research (University of Castilla La Mancha).

Questionnaires were administered in groups of 20–30 students in the assembly room of the high school (with a seating capacity of 350 people), which improved the concentration and privacy of the participants. Guidelines for fulfilling the questionnaires were verbally explained and were also written out before the start of the study. The proctors highlighted the need for the participants to carefully review each item and give sincere answers. To avoid social desirability in the answers, researchers insisted on the fact that the questionnaires were anonymous. The average duration of questionnaire fulfillment was 20 min, but the participants were given 25 min to complete the questionnaire. Once the questionnaires were completed, the students were led one by one to an adjoining room where they were weighted and measured by two expert researchers. During this process, the privacy and confidentiality of their data were similarly guaranteed.

### Data Analysis

Structural equation modeling was used to analyze the data according to a predictive model. All statistical analyses were run using SPSS 24 and AMOS 24 for Windows.

Assumptions of linearity and multicollinearity were examined by inspecting the matrix scatter charts and the condition and variance inflation factor (VIF) indexes. These were obtained by using a regression analysis, with GSC as the dependent variable. The Durbin-Watson statistic was 1.88, so the variables were independent. All condition indexes were less than 7.34, except for BD (16.85), and the higher VIF value was 1.87; therefore, multicollinearity could be discarded. Finally, univariate kurtosis (range: −0.89 and 0.57) and skewness (range: −1.26 and 1.16) were examined. Although their values were not extreme, multivariate assumption was not strictly met. Nevertheless, Mardia’s coefficient of multivariate kurtosis (14.95) was far from the cut-off of 70, so the use of maximum likelihood (ML) as a method of estimation was appropriate (Rodríguez and Ruiz, 2008). ML is robust to the non-normality, especially since the sample size is large (Iacobucci, 2010). To deal with this strict non-normality, SEM analysis was completed with a 1,000 replication bootstrap with a 95% bias-corrected confidence interval.

The proposed initial model and the subsequent ones included BMI and weekly minutes of PA as observable variables, while BD, GPSC, and GSC were considered latent variables with the items of the scales as their estimators. Concretely, the BD had the sum of the 34 items of BSQ as the only one indicator for this latent variable. Therefore, to achieve an identified initial model, the error’s variance of the BSQ had to be estimated from its internal consistency coefficient and its variance by following Kline’s (2011) suggestions. The initial model contained all theoretically possible paths (later explained) and, from this model, different nested models were proposed by following a trimming model process (Kline, 2011).

To test the fit of the models, chi-squared (χ^2^), chi-squared by degrees of freedom ratio (χ^2^/*df*), goodness of fit index (GFI), and root mean square error of approximation (RMSEA) were used as absolute fit indexes. Incremental or comparative fit indexes were also considered by including the Tucker-Lewis index (TLI) and the comparative fit index (CFI). Finally, regarding the parsimony-adjusted index, the Akaike’s information criterion (AIC) and normed fit index (NFI) were used. A χ^2^/*df* ratio < 3 was considered as acceptable (Iacobucci, 2010). Values >0.90 were considered as acceptable and > 0.95 as optimal for the NFI, TLI, GFI, and CFI indexes; for the RMSEA, the cut-off values were < 0.5 or < 0.6 as good and < 0.08 as acceptable (Hu and Bentler, 1999; Schumacker and Lomax, 2010).

## Results

### Participants

The characteristics of the participants are shown in Table 1. They had an age range of 12–17 years, with a mean age of 14.59 (SD = 1.51). The weekly minutes of PA of the males was triple the time of PA undertaken by the females (♂ *M* = 281.46, SD = 203.19; ♀ *M* = 90.45, SD = 137.10). Both the GPSC and GSC were high among the males and significantly greater than among the females (*p* < 0.001). Similarly, the females were more concerned about their bodies than the males (*p* > 0.001), reaching a mean score of 85.14, which exceeded the cut-off value for body image concern of BSQ (>81).

**Table 1:** Characteristics of the participants.

	Male (*n* = 284)		Female (*n* = 350)		All (*n* = 634)		
	*M*	SD	*M*	SD	*M*	SD	*p*
Age	14.50	1.51	14.66	1.50	14.59	1.51	0.177
BMI	22.36	3.93	21.81	3.64	22.05	3.78	0.069
PA minutes per week	281.46	203.19	90.45	137.10	176.01	194.57	0.000**
GPSC	23.88	5.26	19.67	6.49	21.56	6.32	0.000**
GSC	24.66	4.38	22.30	5.10	23.36	4.93	0.000**
BD (BSQ)	54.41	26.80	85.14	39.99	71.37	37.91	0.000**

BMI, body mass index; PA, physical activity; GPSC, general physical self-concept; GSC, general self-concept; BD, body dissatisfaction. M, mean; SD, standard deviation. *p < 0.05; **p < 0.01; ***p < 0.001.

### Structural Models

**Figure 1:**
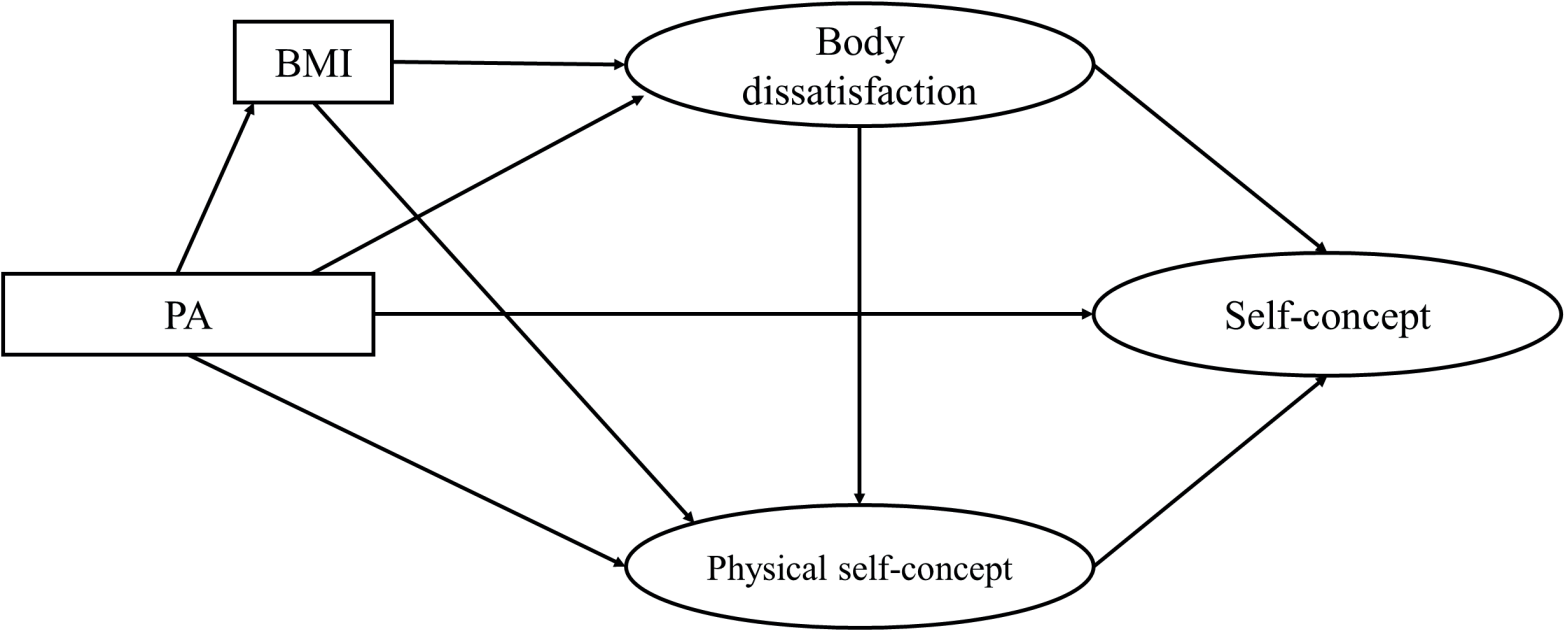
Initial theoretical model (model A1).

The initial model (model A1; Figure 1) starts at the integration of the following theories and models:

1. The EXSEM model (Sonstroem and Morgan, 1989), which established the GPSC as a mediator between the effects of PA on GSC.
2. The operationalization of the EXSEM, which highlights that the effect of PA on GSC is mediated by body satisfaction and physical perceptions (Martin-Ginis et al., 2012, 2014).
3. The model hypothesized by Fox and Corbin (1989), which reflects the multidimensional and hierarchical nature of physical self-concept by placing the perceptions of physical attractiveness as one of its dimensions. Considering body image as a dimension of physical self-concept (Fernández-Bustos et al., 2015), body satisfaction should be a mediator between PA and physical self-concept.
4. The BMI as an exogenous self-contained variable influencing body satisfaction.
5. The model of Mak et al. (2016) suggests that BMI is a mediator between PA and self-esteem.
6. Additionally, for the initial model, the possible direct effects of PA and BD on GSC were included.

**Figure 2:**
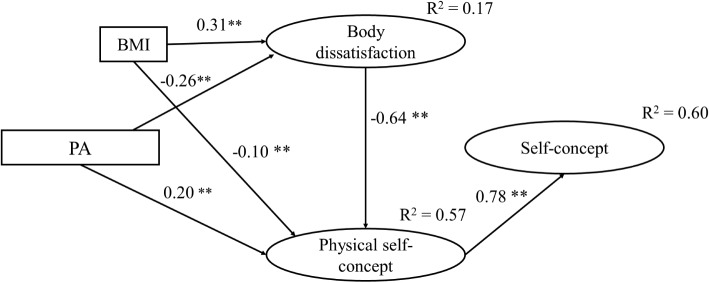
Final theoretical model (model A4) with standardized regression coefficients (betas). *p < 0.05; **p < 0.01.

The initial model fit could be improved (Table 2), as χ^2^/*df* exceeded the established cut-off (χ^2^/*df* = 3.21; GFI = 0.976; RMSEA = 0.059; CFI = 0.974; TLI = 0.956; NFI = 0.963; AIC = 115.48), and four paths reported nonsignificant effects. Therefore, we continued with the model trimming process. First, this study found no relationship between PA and BMI. Since in other studies (Kantanista et al., 2015; Sani et al., 2016) this relationship was also nonsignificant, it was excluded (model A2). This slightly improved the model fit, but the ratio χ^2^/*df* continued to exceed the established threshold. After that, a third model (model A3) was tested by removing the effect of PA on GSC from model A2, which was the next most nonsignificant path. This step was supported by hypotheses holding that this effect is not direct but through the physical self-concept (Sonstroem and Morgan, 1989). This modification also slightly improved the model fit reaching an acceptable value of χ^2^/*df* (2.968). Model A3 did not show a significant effect of BD on GSC. The nonexistence of this path would support the multidimensional, hierarchical conceptualization of self-concept, where BI would be a subdimension of GPSC and an influence through the GPSC (Fox and Corbin, 1989). Thus, another model (model A4) that excluded this effect was tested, showing an optimal fit (χ^2^/*df* = 2.90; GFI = 0.975; RMSEA = 0.054; CFI = 0.975; TLI = 0.962; NFI = 0.962; AIC = 111.66) and significant effects for all the included paths (Figure 2; Table 2). After comparing the last two models, no statistical difference was apparent (Δχ^2^ = 1.395, Δ*df* = 1*, p* = 0.237), so we accepted the model A4 as the final model since it showed a better parsimony fit index (AIC = 111.666).

**Table 2:** Fit indexes by model and group.

Model	*χ*^2^	*df*	*χ*^2^/*df*	GFI	RMSEA	CFI	TLI	NFI	AIC
Model A1	67.487	21	3.213	0.976	0.059	0.974	0.956	0.963	115.487
Model A2	67.877	22	3.085	0.976	0.057	0.974	0.958	0.963	113.877
Model A3	68.271	23	2.968	0.975	0.055	0.975	0.960	0.963	112.271
Model A4^*^	69.666	24	2.902	0.975	0.054	0.974	0.962	0.962	111.666
Model M1	49.924	21	2.377	0.960	0.069	0.955	0.924	0.927	97.924
Model M2	50.063	22	2.275	0.960	0.067	0.957	0.929	0.927	96.063
Model M3^*^	50.980	23	2.216	0.960	0.065	0.957	0.933	0.926	94.980
Model M4	57.836	24	2.409	0.954	0.070	0.948	0.922	0.916	99.836
Model F1	40.454	21	1.926	0.974	0.051	0.980	0.966	0.960	88.454
Model F2	40.469	22	1.839	0.974	0.049	0.981	0.969	0.960	86.469
Model F3	40.566	23	1.763	0.974	0.046	0.982	0.972	0.960	84.566
Model F4^*^	40.936	24	1.705	0.974	0.044	0.983	0.974	0.960	82.936
Model F5	44.616	25	1.784	0.972	0.047	0.980	0.971	0.956	84.616

*A, all; M, males; F, females*.

* *Final model*.

Standardized regression coefficients for each proposed direct path in the final model reached significance at *p* < 0.001 level. The strongest direct effects were reported for GPSC on GSC (*β* = 0.78), being positive, and between BD and GPSC (*β* = −0.64), being negative (Table 3). The results also showed that PA positively and directly influenced GPSC (*β* = 0.20*, p* < 0.01) and indirectly through BD (*β* = 0.17*, p* < 0.01). Similarly, PA had a direct effect on BD (*β* = −0.26*, p* < 0.01) and an indirect effect on GSC mediated by GPSC and BD (*β* = 0.29, *p* < 0.01). The BMI had a direct influence on BD (*β* = 0.32*, p* < 0.01) and GPSC (*β* = −0.10, *p* < 0.01) and an indirect effect on GPSC (*β* = −0.15, *p* < 0.01) and GSC (*β* = −0.14*, p* < 0.01). The final model explained 60% of the variance of GSC and 57% of the variance of GPSC.

**Table 3:** Standardized regression coefficients (direct, indirect and total effects). Final models.

	Model A4			Model M3			Model F4		
Path	D	I	T	D	I	T	D	I	T
PA→BD	−0.26**	–	−0.26**	–	−0.08**	−0.08**	−0.09	–	−0.09
PA→BMI	–	–	–	−0.20**	–	−0.20**	–	–	–
PA→GSC	–	0.29**	0.29**	–	0.17**	0.17**	–	0.21*	0.21**
PA→GPSC	0.20**	0.17**	0.37**	0.16**	0.07**	0.23**	0.21**	0.07	0.28**
BD→GSC	–	−0.50**	−0.50**	−0.28	−0.29***	−0.57**	–	−0.51**	−0.51**
BD→GPSC	−0.64**	–	−0.64**	−0.44**	–	−0.44**	−0.69**	–	−0.69**
BMI→BD	0.31**	–	0.31**	0.44**	–	0.44**	0.43**	–	0.43**
BMI→GSC	–	−0.24**	−0.24**	–	−0.36**	−0.36**	–	−0.29**	0.29**
BMI→GPSC	−0.10**	−0.20**	−0.30**	−0.17*	−0.19**	−0.36**	−0.10*	−0.29**	−0.39**
GPSC→GSC	0.78**	–	0.78**	0.65**	–	0.65**	0.74**	–	0.74**

D, direct effects; I, indirect effects; T, total effects; BMI, body mass index; PA, physical activity; GPSC, general physical self-concept; GSC, general self-concept; BD, body dissatisfaction. *p < 0.05; **p < 0.01; ***p < 0.001.

### Structural Models by Sex

The initial model A1 was further analyzed to test whether the relationships between PA and GSC were different between males and females. The same procedure was followed from the initial model, but the samples of male (model M1) and females (model F1) were considered separately. The initial models M1 and F1 showed modest fit indexes for both males and females (Table 2) and included different nonsignificant paths in each group. For this reason, the design of the subsequent models was different for each group, but in both cases, a model trimming process was used.

**Figure 3:**
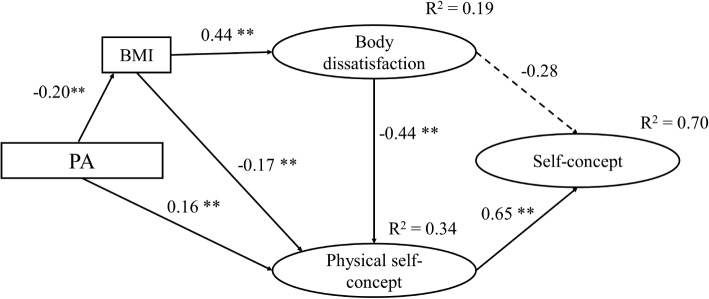
Final model for males (model M3). *p < 0.05; **p < 0.01.

Regarding the male sample, the model M1 included three nonsignificant paths: PA→GSC, PA→BD, and BD→GSC. Therefore, different models were designed by following a procedure similar to the one used with the total sample. Among them, the model M3, which excluded the effects of PA on GSC and BD, showed the best fit (χ^2^/*df* = 2.21; GFI = 0.960; RMSEA = 0.065; CFI = 0.957; TLI = 0.933; NFI = 0.926; AIC = 94.98). This model also included the effect of BD on GSC, whose coefficient was close to the level of significance (*β* = −0.28, *p* = 0.05). Additionally, a fourth model (model M4), which excluded that effect, was tested, but it demonstrated worse fit indexes (Table 2). As there were no statistical differences between M2 and M3 (Δχ^2^ = 0.916, Δ*df* = 1, *p* = 0.338), we opted for model M3 (Figure 3) as the final model for males because it yielded more appropriate RMSEA and AIC fit indexes.

**Figure 4:**
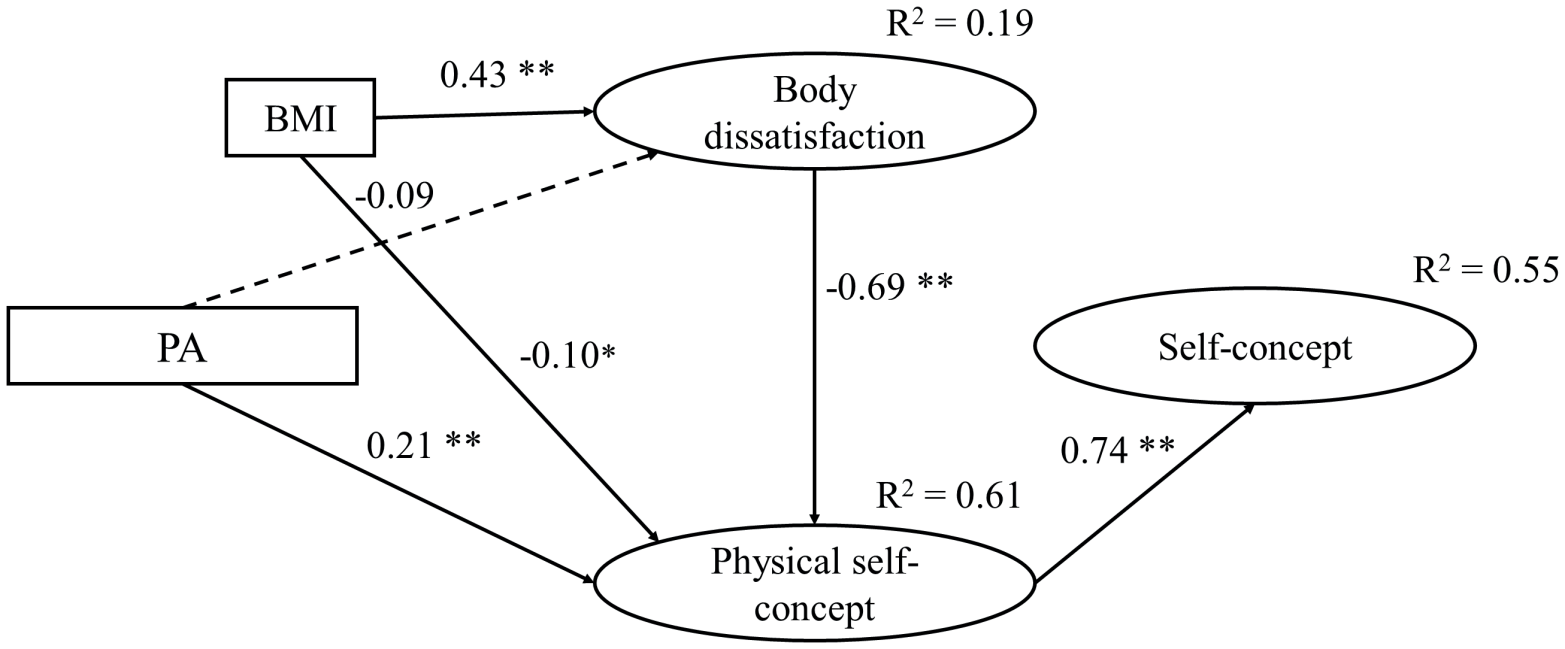
Final model for females (model F4). *p < 0.05; **p < 0.01.

Concerning the female sample, four models were created in addition to model F1 by following a procedure similar to the one used with the total sample. As in model M1 for males, in model F4 for females, the effect of PA on BD was nonsignificant. Thus, we additionally tested a model F5 excluding that effect. However, the fit indexes were less acceptable than those demonstrated by the previous models (Table 2). Model F4 had the best fit (χ^2^/*df* = 1.70; GFI = 0.974; RMSEA = 0.044; CFI = 0.983; TLI = 0.974; NFI = 0.960; AIC = 82.93) and was not statistically different from either model F3 (Δχ^2^ = 0.369, Δ*df* = 1, *p* = 0.543) or model F5 (Δχ^2^ = 3.680, Δ*df* = 1, *p* = 0.055). Thus, model F4 (Figure 4) was selected as the final model for females. This model included the effect of PA on BD, which demonstrated a standardized coefficient very close to significance, but without reaching it (*β* = −0.10, *p* = 0.05).

The main differences between the final models (Table 3) for males (model M3) and females (model F4) were found in the direct effect of BD on GPSC (♂ *β* = −0.44; ♀ *β* = −0.69) and the indirect effect of BD on GSC (♂ *β* = −0.29; ♀ *β* = −0.51). In addition, among males, the path between PA and BMI was also considered. A negative, direct effect was found (*β* = −0.20, *p* < 0.05), placing BMI as a mediator between PA and BD; while among women, only a very little and direct effect of PA on BD was found (*β* = −0.10, *p* = 0.05). The final model for males explained 34% of the variance of GPSC and 70% of the variance of GSC, while for females, the final model explained 61 and 55%, respectively.

## Discussion

The aim of this study was to design a complementary and explanatory model of the relationships between PA and self-concept. For that purpose, BD, which in previous studies had been related to PA, was included in the model as a determining factor in the self-concept configuration of adolescents. GPSC was also included as a mediating variable. BMI was finally added, assuming its influence on PA, BD, and GPSC. Additionally, we aimed to know whether it was possible to yield different models distinguishing by sex. This was especially necessary as it was previously evidenced a higher physical self-concept for males (Hagger et al., 2005) and no clear moderating role of sex appears to exist in the relationship between PA and BI (Bassett-Gunter et al., 2017). This situation is evident from the different influence of the aesthetic model in men and women, which is internalized early, and especially affects females with the impetus to be thin. This makes female adolescents feel much more dissatisfaction with their bodies than their male peers (Grogan, 2006; Kantanista et al., 2015) and to have lower levels of self-esteem (Robins and Trzesniewski, 2005), as was found in the participants of the present study.

For the design of the initial model, we considered the theory about the effect of PA on self-concept and the effect of PA on BI. We started from an adaptation of EXSEM (Sonstroem and Morgan, 1989) and some aspects from the variant proposed by Martin-Ginis et al. (2012) or the role of BMI on PA and BI (Mak et al., 2016). The possible direct effects among some variables, such as PA and GSC (Sani et al., 2016), were also included. From the initial model, other different models were proposed, and those that yielded the best fit indexes and theoretically coherent were chosen as the final models. In any case, even though the most relevant theoretical aspects coincided, the final models differed between males and females.

In all cases, the important positive effect of PA on the self-concept of adolescents was confirmed, as has been seen in previous studies (Beasley and Garn, 2013; Liu et al., 2015) with no sex differences (Reddon et al., 2017). The suggested models explained more than 50% of the variance of GSC. However, the effect of PA on GSC was not direct but through one of the dimensions of self-concept, the physical self-concept (Sonstroem, 1997). Thus, in line with other studies that included the physical self-concept as a mediating variable, both the hierarchical and multidimensional conception of self-concept (Shavelson et al., 1976) and the EXSEM model are confirmed. Previous studies found a direct association of PA with self-concept but without including physical self-concept in the model (Sani et al., 2016).

Within this hierarchical model, working on GPSC, or some of its subdomains, would be easier than working on the GSC itself. The former’s effect on the latter will be based on the importance that each subject confers to the physical domain (Zulaika, 2002). During adolescence, physical self-concept takes on special significance due to the importance conferred to everything that is related to the physical domain since this dimension is one of the most influential factors on the self-concept configuration in youths (Harter, 1999). In this study, that situation can be seen in the emphasized effect of GPSC on GSC in all models, being slightly more important for females.

The most well-known models explaining the physical self-concept (Fox and Corbin, 1989; Marsh et al., 1994) include physical appearance as a subdomain of physical self-concept. BI is closely related to the physical attractiveness domain, understanding the latter as one’s perceptions about his/her physical appearance and his/her satisfaction and confidence with his/her self-image (Goñi et al., 2004). Therefore, BI would be a part of physical self-concept (Burns, 1990; Fernández-Bustos et al., 2015). Despite the link between BI and physical self-concept, there are only a few studies that show the relationship between them. The model presented in this study proves the large direct effect of BI on GPSC in adolescence, along with the effect on GSC, which are both very high and indirect. This supports the hierarchical and multidimensional model previously explained. In this way, many studies highlight the important relationship between BD and self-esteem in both males and females (Van den Berg et al., 2010; Fernández-Bustos et al., 2015).

Considering the lack of physical self-concept subdomains other than BI in the suggested model, PA had a positive and direct effect on GPSC, as was supported in all the previous literature, which is reinforced by the results of the meta-analysis carried out by Babic et al. (2014). Similarly, following the hierarchical model, PA indirectly influenced GPSC through its subdimension of BI, as measured by BD.

Regarding the effect of PA on BD, the three final models yielded different results. For all participants, the positive and direct effect of PA on body satisfaction was confirmed (Añez et al., 2018); this effect, however, was more relevant in females than males, which is consistent with Hausenblas and Fallon’s (2006) findings. In the case of males, there was no direct effect of PA on BD, but it was mediated by BMI.

BMI was shown as a variable of especial interest in the suggested models. On the one hand, BMI proved that it plays a determining role in body satisfaction (Strauss, 2000), having a negative effect on both males and females. Indeed, high adiposity is thought to be a promoter of body dissatisfaction, as it is opposed to the aesthetic model of males and females. Therefore, the larger the deviation degree from the ideal physical shape, the greater the dissatisfaction with the resulting body (Stice and Shaw, 2002). On the other hand, BMI demonstrated negative direct and indirect effects on the perceptions of GPSC and an indirect effect on GSC. Several studies agreed that there was a relationship between obesity and lower self-esteem and worse physical perceptions (Griffiths et al., 2010; McClure et al., 2010). Altıntaş et al. (2014) considered BMI to be the best predictor of self-esteem and body satisfaction in female adolescents, being slightly less important among males. Sani et al. (2016) tried to create a model to explain the relationships between PA, BI, and self-esteem, but they only found a direct association of BMI with BI and no association with self-esteem.

Nevertheless, even though some studies associated PA with lower values of BMI (Du et al., 2013), in the present study, this was only evidenced in males, in accordance with Kantanista et al. (2015). This result can be explained in view of some evidence among females that associated a higher BMI with higher PA levels triggered by their desire to exercise to lose weight (Ingledew and Sullivan, 2002). Additionally, the possible mediating role of BMI in the relationship between PA and GSC, already hypothesized in previous studies (Reddon et al., 2017), was only supported among males, which is in line with Mak et al. (2016).

In practice, the results of this research should help to explain how the effect of PA on students’ BI and self-concept is produced and to place value on the promotion of PA in educational centers. By doing that, educational policymakers will contribute to the development of not only the physical health of adolescents but also their psychological well-being at an educational stage of special difficulty, where PA tends to decrease alarmingly in comparison to early stages. These results are also relevant for Physical Education teachers to stress the importance of optimizing the time of PA during their lessons; the results can be also useful for providing a Quality Physical Education that makes students improve their physical perceptions, find pleasure in exercise, and achieve autonomous active lifestyle behaviors outside of school.

This study presents some limitations that must be taken into consideration. Among these, the assessment of a unique aspect of BI – the dissatisfaction in terms of weight loss – must be pointed out. This is particularly relevant for males, who presented an aesthetic model that tends to drive for muscularity and away from obesity. That is, BD in terms of desiring a more muscular body has been omitted, and this especially influences males. Another limitation would be the lack of more indicators of PA, in addition to the weekly minutes, as the type of activity (endurance, strength, etc.) may determine the perceptions of BI and the physical self-concept itself.

It is necessary to continue with the study of the moderating effect of sex in the relationship between PA and BI. It is also necessary to examine the potential moderators that are exclusive to male or female samples in future research and interventions. To do so, besides taking into account the differences in the configuration of the body image in both sexes, there is a need to include variables that determine the relationship between PA and BI, such as the aesthetical reasons that lead to the practice of PA or the effect of different types of physical exercises or sports on BI.

## Conclusion

To conclude, the suggested model put emphasis on how PA is associated with a more positive self-concept during adolescence, although this effect is mediated by both physical self-concept and BI. Likewise, PA is associated with a lower BD, while BMI is shown as an important external variable in the model, whose role varied by sex. The importance of body satisfaction and physical self-concept in the configuration of GSC in adolescents is also highlighted, especially among females.

## Data Availability

The raw data supporting the conclusions of this manuscript will be made available by the authors, without undue reservation, to any qualified researcher.

## Ethics Statement

Prior to data collection, we informed the participants and their parents about the research aims, procedures, risks, and benefits of the study, and we obtained the required consent from the corresponding educational boards, high schools administrations, and parents of the students. Participation was voluntary with the parents’ consent, and the anonymity of the participants and data confidentiality were guaranteed during the whole process. The study conformed to the deontological guidelines defined by the Declaration of Helsinki (Hong-Kong revision, 1989) and followed the recommendations of the Good Clinical Practice in the European Community (1 July 1991, document 111/3976/88) and the Spanish legislation on human clinical research (Royal Decree 561/1993 on clinical trials).

## Author Contributions

JGF-B designed the study, collected the data, wrote the article, and collaborated on the data analysis. AI-P analyzed the data and collaborated on the article wording. RC collaborated on the article wording and review. ORC designed the study and collaborated on the review of the article.

### Conflict of Interest Statement

The authors declare that the research was conducted in the absence of any commercial or financial relationships that could be construed as a potential conflict of interest.
